# Single‐cell spatial transcriptomic and proteomic characterization of a novel mouse model of late‐onset AD

**DOI:** 10.1002/alz.086723

**Published:** 2025-01-03

**Authors:** Nellie Kwang, Kim N. Green, Vivek Swarup

**Affiliations:** ^1^ Institute for Memory Impairments and Neurological Disorders (MIND), Irvine, CA USA; ^2^ University of California, Irvine, Irvine, CA USA

## Abstract

**Background:**

Late‐onset Alzheimer’s disease (LOAD) represents the majority of human AD cases, yet the availability of animal models that accurately reflect LOAD progression and pathology is limited. Traditional transgenic mouse models including 3xTg‐AD and 5xFAD rely on supraphysiological overexpression of familial AD risk genes, failing to adequately replicate the disease progression observed in LOAD. Here, we present the first characterization of MODEL‐AD1 (MAD1), a platform mouse developed by the Model Organism Development and Evaluation for Late‐onset Alzheimer’s Disease (MODEL‐AD) Consortium. MAD1 features replacements of murine Aβ (*App*), *Mapt*, and *Apoe* alleles with their human counterparts hAβ, *hMAPT* (H2), and *hAPOE4*. These humanized alleles are regulated similarly to their murine equivalents and expressed at physiological levels.

**Method:**

Brain sections from 4‐month‐old (mo) MAD1 and B6J (WT) (n = 3/sex/group) mice were analyzed using NanoString CosMx single‐cell hybridization‐based spatial transcriptomics and antibody‐based spatial proteomics to probe region‐specific differences in gene expression and protein distribution. Further, to examine the role of demyelination in AD pathology, snRNA‐seq analysis was performed on hippocampal and cortical samples of 8‐mo MAD1 mice treated with cuprizone (n = 3M,2F/group), a copper chelator that causes oligodendrocyte degeneration and myelin loss. Finally, snRNA‐seq findings were validated with immunohistochemistry by staining for glial and AD markers.

**Result:**

Spatial transcriptomic and proteomic analyses revealed distinct differences in gene expression and protein distribution between MAD1 and WT mice. Key genes involved in neuroinflammation, synaptic function, and neuronal survival showed altered expression in MAD1 mice, indicating a shift towards an AD‐like state. Congruent with our spatial analyses, snRNA‐seq analysis revealed increased glial cell activation in MAD1 compared to WT. Additionally, 8‐mo MAD1 mice treated with cuprizone exhibited marked microgliosis and astrogliosis compared to MAD1 controls, as demonstrated by immunohistochemistry and snRNA‐seq. These pronounced glial changes were not sufficient to induce Aβ or tau formation at this stage.

**Conclusion:**

These exciting preliminary findings establish MAD1 as a novel platform model for testing various interventions in AD research, including demyelination. Future studies will explore the effects of aging, diet, remyelination, and other factors on MAD1 mice to elucidate the mechanisms of LOAD.

